# Supplementary material for: Probing the Mechanical Properties of DNA Nanostructures with Metadynamics
Source: ACS Nano. 2022 May 17;16(6):8784–97. doi: 10.1021/acsnano.1c08999 (PMC9245350; doi:10.1021/acsnano.1c08999)
Supplement: Supplementary file 1 — nn1c08999_si_001.pdf [file nn1c08999_si_001.pdf]

Probing the mechanical properties of DNA nanostructures with metadynamics: SI

Will T. Kaufhold,[*,†,‡] Wolfgang Pfeifer,[¶] Carlos E. Castro,[¶] and Lorenzo Di Michele[*,‡,†,§]

†*Department of Physics, University of Cambridge, JJ Thomson Avenue, Cambridge CB3 0HE, UK*

‡*Department of Chemistry, Molecular Sciences Research Hub, Imperial College London, London W12 0BZ, UK*

¶*Department of Mechanical and Aerospace Engineering, The Ohio State University, Columbus, OH 43210, USA*

§*fabriCELL, Molecular Sciences Research Hub, Imperial College London, London W12 0BZ, UK*

E-mail: willtkaufhold@gmail.com; l.di-michele@imperial.ac.uk

Supplementary Notes

Supplementary Note 1: MetaD applied to a simple 1D walker

To illustrate the principle of metadynamics here we consider a 1D walker of unit mass moving with Langevin dynamics in a bistable potential as shown in figure S1**a**. For convenience, $k_B T$ is set to 1. The underlying potential energy landscape is:

$$\begin{aligned} U(x) = &- A_1 \cdot e^{-(x-x_1)^2/2\sigma^2} - A_2 \cdot e^{-(x-x_2)^2/2\sigma^2} \\ &+ A_3 \cdot x^2. \end{aligned} \tag{S1}$$

Here, $A_1 = A_2 = 10$, $A_3 = 0.01$, $x_1 = -10$, $x_2 = 10$, $\sigma = 3$.

Langevin dynamics were evaluated by Euler integration:[1]

$$\begin{aligned} x_{\upsilon+\Delta\upsilon} &= x_\upsilon + \dot{x}_\upsilon \cdot \Delta\upsilon \\ \dot{x}_{\upsilon+\Delta\upsilon} &= \dot{x}_\upsilon - \gamma\, \dot{x}_\upsilon \cdot \Delta\upsilon \quad + \sqrt{2\gamma}\, \Delta W_\upsilon - \frac{dU(x)}{dx} \cdot \Delta\upsilon. \end{aligned} \tag{S2}$$

Here $\Delta\upsilon$ is the integration step, while the subscript υ denotes time. The linear damping coefficient is set to $\gamma = 10$. ΔW_υ is a delta-correlated noise, chosen independently at each time step from a normal distribution with variance $\Delta\upsilon$, centre 0. $U(x)$ and its numerical derivative were evaluated through linear interpolation on a grid. The integrator used a timestep of $\Delta\upsilon = 0.01$. The metadynamic parameters were $A = 0.5$, $\sigma = 1$, $\tau = 1000$, $\Delta T = 5$.

Figure S1**b** (top) shows a conventional MD trajectory. A configuration initialized in one minimum cannot cross to the other, as doing so would require passing through an unlikely transition state. The histogram approach of evaluating free energies will therefore only describe a narrow set of conformations as the existence of the unsampled free energy minimum cannot be inferred from the trajectory.

MetaD flattens the free energy landscape, enabling otherwise unlikely transitions between local minima as illustrated in figure S1**b** (bottom). Although initially the particle is trapped in one well, it subsequently transitions to the other, before starting to reversibly visit both wells with a diffusive motion, unaffected by the potential barrier. This time-dependent shift in dynamics is enabled by an history-dependent bias potential, built as discussed in the main text (equations 1-3).

For our simple example, the time evolution of the learned bias is illustrated in figures S1**c**,**d**. Initially, the system is trapped in one minimum (figure S1**d**, t=100), but it builds up a bias which allows exploration of the potential energy well. Eventually the potential landscape experienced by the particle (from combining bias and true free energy) no longer prevents access of the transition state.

Equation 3 in the main text shows how, for well-tempered MetaD, B_t converges to a fraction of the true free energy. For an illustration of this convergence, consider the time evolution of the uncorrected potential, $B_t + U$, in our simple example (figure S1**e**). Although initially, the uncorrected potential is U, it subsequently becomes flattened by the bias, converging to $\frac{T}{T+\Delta T}U$ (cnf main text, noting that in this 1D example $U \equiv \Delta G$). Analogously, figure S1**f** shows convergence of $\frac{\Delta T+T}{\Delta T}B_t$ to U. MetaD therefore provides a method to acquire free energy profiles from the converged bias, even in systems with high free energy transition states.

Supplementary Note 2: Isomerization in a re-configurable bistable tile

Base stacking interactions have previously been used to create reconfigurable devices,[2] and a similar approach has recently been applied to design origami-based reconfigurable molecular arrays, which can spatially relay information through the propagation of conformational transitions.[3] The elementary unit of the array is a bistable motif, in which transition between the two configurations requires stacking interactions to break. These elements can be tessellated, creating a rectangular array that preserves the bistability of the individual unit. As a further case-study, here we use our oxDNA MetaD approach to map the bistable free energy landscape of this elementary motif.

Figure S5**a** shows the structure under consideration, which is a slightly truncated version of the unit used in bistable origami.[3] In our implementation, each of the shorter strands is 17 nts long (1.5 helical turns), against 21 nts in the origami (2 helical turns).[3] Additionally, in experiments, the longer strand would have a strand break at some location – however, to maintain symmetry, it is circular in the studied model. We do not expect either of these features to have a qualitative effect on the free energy landscape.

When simulated under unbiased MD, the structure remains trapped in a single conformer. A two dimensional reaction coordinate is defined corresponding to the distances x_1 and x_2 between centers of mass of the four nucleotides adjacent to each strand break, as illustrated in figure S5**a**. In a single conformer, one of these values takes a higher value (the helical rise of 34 bps $\approx$ 11.5 nm), while the other takes a lower value (slightly over the width of a helix $\approx$ 2.1 nm).

MetaD simulation along this collective variable constructs a bias which enables frequent transitions between the two free energy minima, and can be used to extract the free energy landscape shown in figure S5**b** (left). Demonstrations of convergence are illustrated in figure S6. The symmetry of the free energy landscape structure was not externally imposed, and emerges simply from the multiple transitions between energetically similar conformers.

One of the disadvantages of a 2D collective variable is the significant simulation time necessary for convergence. The structure of the free energy landscape in this example indicates that one could simply bias by the angle subtended by the x-axis and a line from the origin to (x_1, x_2), *i.e.* $\theta = \arctan \frac{x_2}{x_1}$, as plotted in figure S5**b**. This enables a faster convergence, and acquisition of the landscape illustrated in figure S5**b** (right). Here, biasing is only possible in θ, so that investigation into the free energy as a function of r can only be done thermally – hence the structure revealed here corresponds to a small region in the radial coordinate, r, expanded at the minimum for each angular coordinate θ.

The free energy projections in figure S5**b** look deceptively continuous. However, analysis of actual trajectories indicates that the system undergoes transitions between stacking states identified by their coaxial stacking configurations. Since there are four possible stacking locations, each of which can be either stacked or unstacked, there are a total of 16 states. Geometrical requirements eliminate essentially all of these configurations except those illustrated in figure S5**c**, which show the transition path followed at early times in both 1D and 2D metadynamics. A single coaxial stack detaches, which is followed by transition into an entirely unstacked intermediate, the formation of one of the stacks in the alternative conformer, and finally that of the opposite stack.

However, we note that great care must be taken in interpreting these transitions as representative of the spontaneous transition path of the system, as they are strongly influenced by the reaction coordinate and the accumulating bias. While looking at early simulation times mitigates against the effect of biasing, accurate assessment of transition mechanisms must be performed using inherently dynamical rare event simulation approaches such as Forward Flux Sampling.[4]

Supplementary Figures

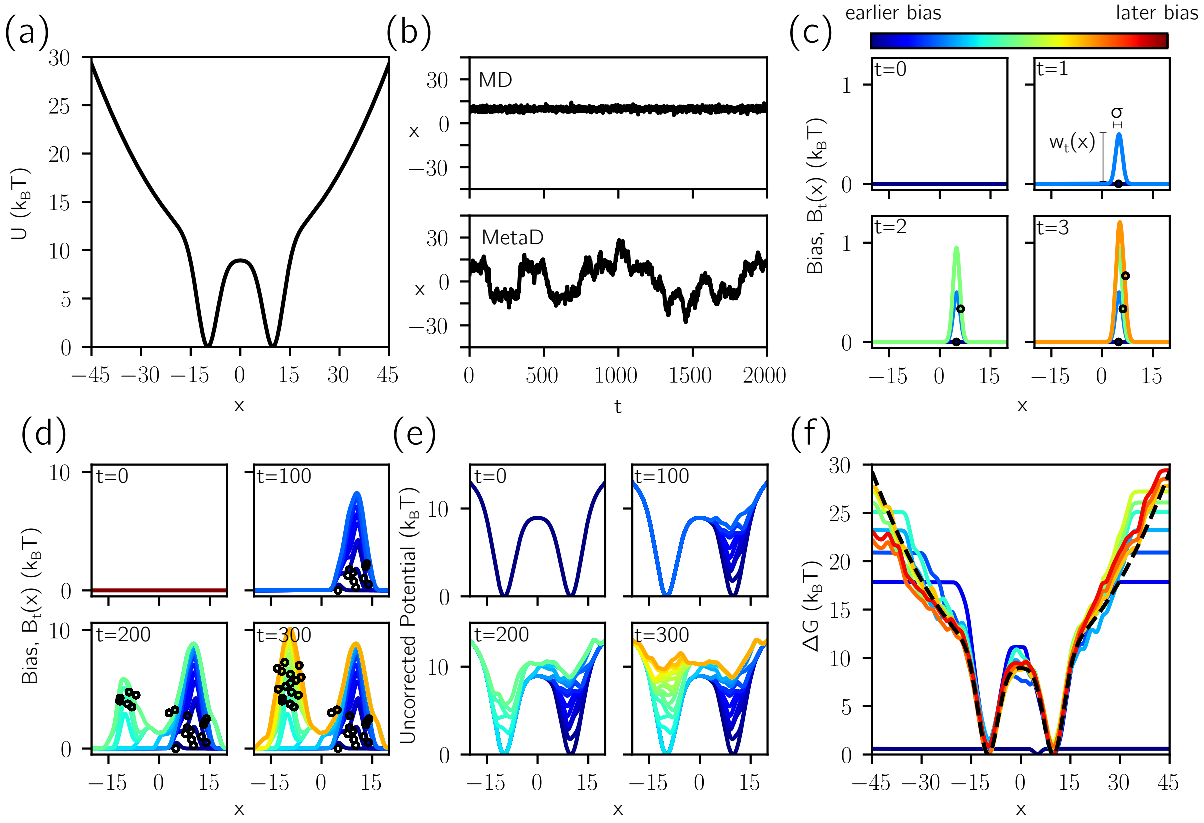

Figure S1: A 1D landscape illustrating the principle of metadynamics. **(a)**, Consider a particle moving with stochastic dynamics on the 1D potential energy landscape illustrated. Here, two potential energy minima are separated by a $\approx 10\,k_BT$ transition state. **(b)**, The dynamics of MD on this landscape are non-ergodic; after initialization in one potential energy minimum, the particle cannot escape in the timescale of simulation. By contrast, MetaD learns to escape the first minimum after 200 iterations of metadynamics. (Note that for consistency with the literature, t refers to the number of iterations of metadynamics, not the number of timesteps.) **(c)**, Metadynamics learns a history dependent potential, $B_t(x)$, whose update is illustrated here. The bias is initialized to 0 ($t = 0$). The position of the particle is illustrated by the black circle. A repulsive Gaussian potential is added at $t = 1$; this has height $w_t(x)$ and standard deviation σ. The system is then evolved under the action of both the potential, $U(x)$, and the bias, $B_t(x)$. **(d)**, Bias evolution at longer timescales: black circles indicate positions, where the higher the point, the more recent. **(e)**, The uncorrected (residual) potential felt by the system: $U(x) + B_t(x)$. Although initially the uncorrected potential is bistable, at $t = 300$ it has largely been corrected to an almost flat landscape. **(f)**, The free energy (U for this trivial particle) can be estimated by $\frac{\Delta T+T}{\Delta T}B_t(x)$ after the bias converges. The true landscape here is illustrated by a dashed black line, and the time evolving free energy implied by the bias by colored lines.

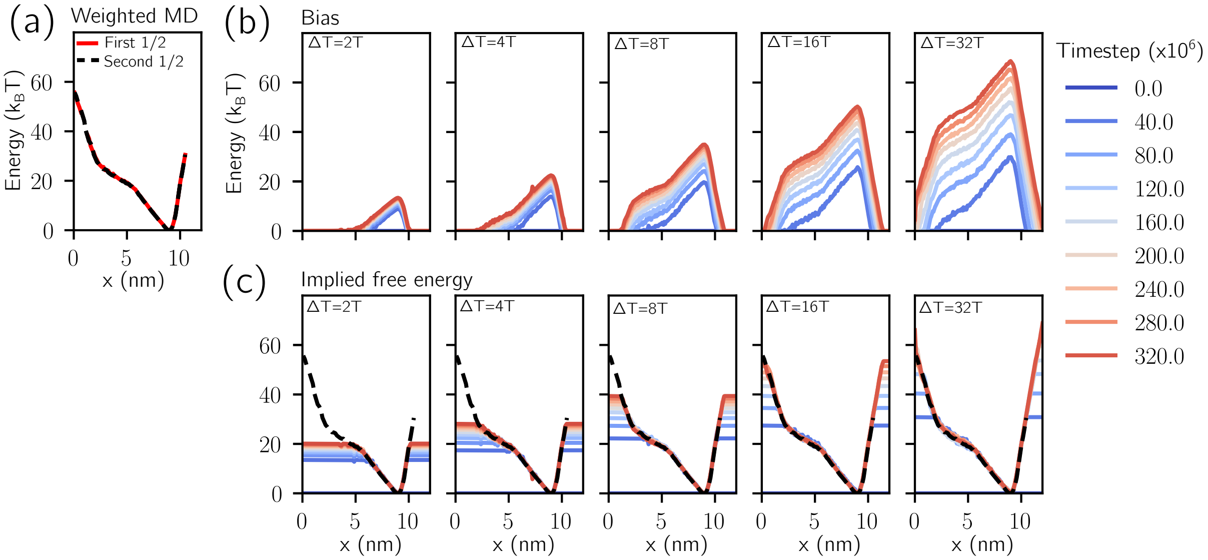

Figure S2: Demonstrations of convergence in dsDNA buckling. **(a)**, the free energy as a function of x from biased MD simulation – results inferred from the first and second halves of MD simulation are plotted indicating convergence. **(b)**, time-dependent growth of the bias potential for various values of ΔT. **(c)**, Free energies implied from MetaD, overlaid with the known free energy from weighted molecular dynamics (black dashed line). The legend, applying to panels **c** and **d** indicates MD time-steps from the start of the simulation.

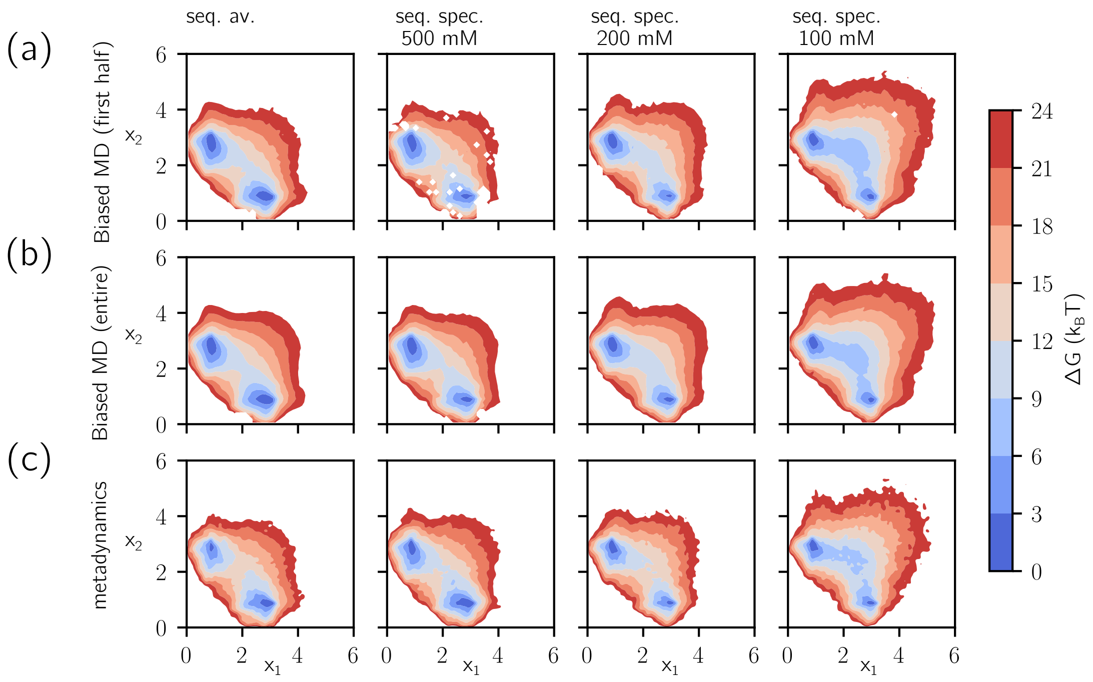

Figure S3: Demonstrations of convergence in Holliday junction isomerization. **(a)**, Free energy distribution estimated by a histogram of biased MD simulation during the first half of simulation. **(b)**, Distributions of the same, but over the entire simulation period. **(c)**, Implied free energies from metadynamics simulation. The slightly speckled structure is a consequence of the small σ used in metadynamics.

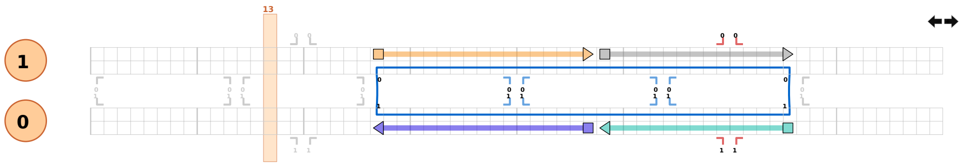

Figure S4: caDNAno routing of the bistable unit.

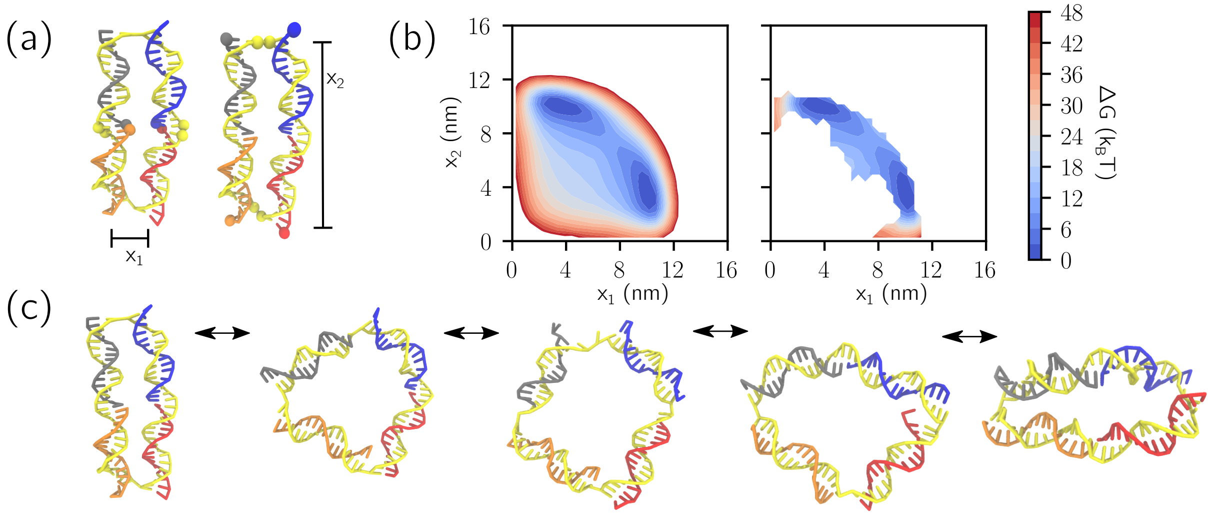

Figure S5: MetaD enables mapping of the free energy landscape of a switchable bistable unit. **(a)**, A snapshot of a likely conformation of the bistable unit. Collective variables x_1 (left), and x_2 (right), are distances between centers of mass of the indicated beads. These were used to bias the dynamics and encourage transitions. **(b)**, (Left) Well-tempered MetaD simulation ($\Delta T = 20T$) provides a bias which flattens the free energy landscape. This enables static bias MD to reconstruct the symmetric bistable distribution expected (convergence of the bias is demonstrated in figure S6). (Right) Biasing can instead be done in one dimension to accelerate convergence – here the angular coordinate $\theta = \tan^{-1}(x_1/x_2)$ is used. This confines the region sampled to a thin shell around the free energy minimum for each value of θ; since a smaller configuration volume is sampled, convergence is faster. **(c)**, A proposed transition mechanism, observed as the first transition between conformers for both the 1D and 2D metadynamics simulations.

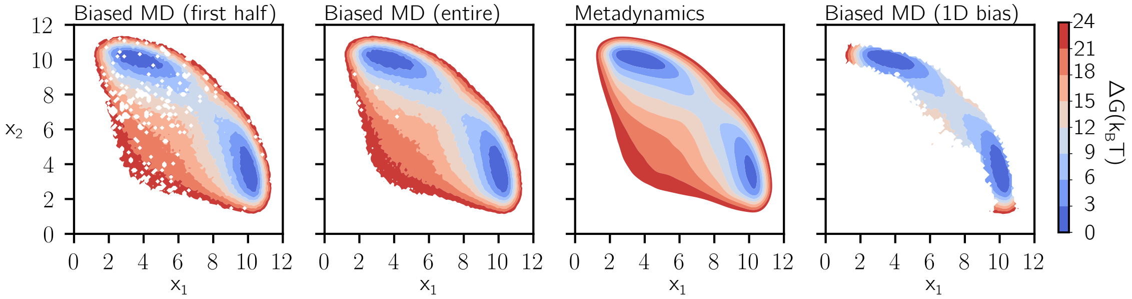

Figure S6: Demonstrations of convergence in bistable unit isomerization. The distribution acquired from the first half of biased MD simulation is essentially identical to that from the entire trajectory, bar some sparsity of sampling. This is similar to that acquired by MetaD, although the latter was not run for sufficiently long to correct the asymmetry in the sizes of the two free energy minima – these should be symmetric due to the symmetry of the molecule. Running a 1D MetaD simulation based on angular coordinate $\theta = \arctan(x_2/x_1)$ enables acquisition only of likely states as a function of this angle – hence the limited region of converged observations. Here, we have plotted the distribution from a MD run with a static 1D bias chosen from the converged MetaD simulation run.

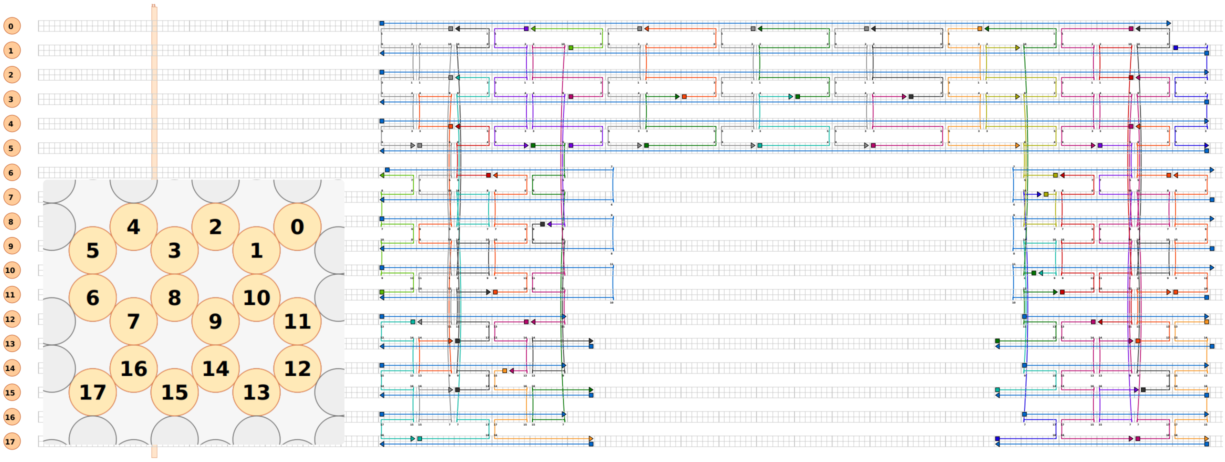

Figure S7: caDNAno routing of the truncated compliant joint.

Supplementary Tables

Table S1: Parameters for MetaD simulations. $X_{\min}$ and $X_{\max}$ indicate the limits of the range accessible to the collective variables. The meaning of all other parameters is described in the main text. "ox." indicates oxDNA units.

System	A (k_BT)	σ (ox.)	τ (steps)	$\Delta T/T$	$X_{\min}$ (ox.)	$X_{\max}$ (ox.)	δx
dsDNA	0.5	0.05	4×10^5	2,4,8,16,32	0	20	0.005
Bistable	0.5	0.5	4×10^5	20	-1	30	0.1
Bistable (1D)	0.5	0.01	2×10^4	20	-1	1.7	0.002
HJ	1	0.1	1×10^5	20	-0.1	10	0.005
Compliant joint	1	2	1×10^5	32	0	80	0.1

Table S2: Numbers of timesteps and replicas for MetaD simulation and biased MD simulation.

System	N_{steps} (MetaD)	N_{replicas} (MetaD)	N_{steps} (MD)	N_{replicas} (MD)
dsDNA	300×10^6	32	1×10^9	105
Bistable	200×10^6	32	230×10^6	48
Bistable (1D)	190×10^6	6	190×10^6	48
HJ (seq. av.)	355×10^6	6	500×10^6	48
HJ (500 mM)	260×10^6	6	500×10^6	48
HJ (200 mM)	355×10^6	6	400×10^6	48
HJ (100 mM)	280×10^6	6	250×10^6	48
Compliant joint	3×10^7	4	1×10^7	24

Table S3: Sequences of the truncated J3 Holliday junction (5' → 3').

**Name**	**Sequence**
**A**	CGGTAGCAGCC TGAGCGGTGGT
**B**	ACCACCGCTCA ACTCAACTGCA
**C**	TCCTAGCAAGG GGCTGCTACCG
**D**	TGCAGTTGAGT CCTTGCTAGGA

References

1. Kloeden, P. E.; Platen, E. *Numerical Solution of Stochastic Differential Equations*; Springer Berlin Heidelberg, 1992.

2. Gerling, T.; Wagenbauer, K. F.; Neuner, A. M.; Dietz, H. Dynamic DNA devices and assemblies formed by shape-complementary, non-base pairing 3D components. *Science* **2015**, *347*, 1446–1452.

3. Song, J.; Li, Z.; Wang, P.; Meyer, T.; Mao, C.; Ke, Y. Reconfiguration of DNA molecular arrays driven by information relay. *Science* **2017**, *357*.

4. Allen, R. J.; Valeriani, C.; Rein Ten Wolde, P. Forward flux sampling for rare event simulations. *Journal of Physics Condensed Matter* **2009**, *21*.
